# Targeting ADRB2 enhances sensitivity of non-small cell lung cancer to VEGFR2 tyrosine kinase inhibitors

**DOI:** 10.1038/s41420-022-00818-8

**Published:** 2022-01-24

**Authors:** Yingzhuo Xu, Jian Wang, Xu Wang, Xiaoshu Zhou, Jing Tang, Xiaohua Jie, Xijie Yang, Xinrui Rao, Yunhong Xu, Biyuan Xing, Zhenyu Li, Gang Wu

**Affiliations:** Cancer Center, Union Hospital, Tongji Medical College, Huazhong University of Science and Technology, Wuhan, 430022 China

**Keywords:** Targeted therapies, Non-small-cell lung cancer

## Abstract

Vascular Endothelial Growth Factor Receptor 2 (VEGFR2) tyrosine kinase inhibitors (TKIs) have achieved remarkable clinical progress in the treatment of non-small-cell lung cancer; however, resistance has limited their therapeutic efficacy. Therefore, understanding the mechanisms of VEGF-TKI and ICI resistance will help to develop effective treatment strategies for patients with advanced NSCLC. Our results suggested that treatment with VEGFR2-TKIs upregulated ADRB2 expression in NSCLC cells. Propranolol, a common ADRB2 antagonist, significantly enhanced the therapeutic effect of VEGFR2-TKIs by inhibiting the ADRB2 signaling pathway in NSCLC cells in vitro and in vivo. Mechanically, the treatment-induced ADRB2 upregulation and the enhancement of ADRB2/VEGFR2 interaction caused resistance to VEGFR2-TKIs in NSCLC. And the inhibition of the ADRB2/CREB/PSAT1 signaling pathway sensitized cells to VEGFR2-TKIs. We demonstrated that ADRB2 signaling is crucial in mediating resistance to VEGFR2-TKIs and provided a novel promising combinatory approach to enhance the antitumor effect of VEGFR2-TKIs in NSCLC combining with propranolol.

## Background

Lung cancer is a leading cause of cancer-related deaths worldwide [1], with non-small cell lung cancer (NSCLC) a major type that accounts for 80–85% of all lung cancers. Unfortunately, 75% of NSCLC patients have advanced-stage or metastatic disease at the time of diagnosis [2].

New blood vessel formation is a complex regulatory process that can promote the occurrence, development, and distant metastasis of malignant tumors, including lung cancer [3]. The tumor vascular network provides essential nutrients and oxygen for tumor cells and removes waste products [4].

Anti-angiogenesis targeted therapy helps treat various cancers and has gradually developed into an effective tumor therapy approach [5]. The VEGF and VEGF receptor (VEGFRs) axis triggers multiple signal networks that eventually result in angiogenesis, cell proliferation, migration, and invasion [6]. In the VEGFR family, VEGFR-2 is most closely associated with angiogenesis [7]; therefore, blocking VEGFR-2 could benefit malignant tumor treatment [8].

VEGFR2-tyrosine kinase inhibitors are anti-tumor efficient; they block VEGFR2, inhibiting tumor angiogenesis [9]. Targeting the angiogenic process is clinically beneficial, but it also has its limitations. Antiangiogenic therapy has only been moderately effective on overall survival (OS) [10], and different TKIs have not been consistently reliable in treating advanced NSCLC [11]. Of major concern is the inability to translate progression-free survival (PFS) and patient response rate benefits into improved OS during VEGFR-TKI monotherapy [12]. Perhaps blocking one angiogenic pathway upregulates other compensatory pathways [13, 14]; if so, then an effective prognostic marker must be identified urgently, and effective combination therapy should be assayed.

β_2_-adrenergic receptor (ADRB2) is a member of the superfamily of G protein-coupled receptors (GPCRs), which are prototypic and ubiquitous cell-surface proteins [15]. This particular receptor has recently drawn increased scrutiny to its role in tumor regulation. One study reported finding ADRB2 activation by isoprenaline (ISO) in NSCLC cells to increase VEGF expression [16]. Mechanistically, ADRB2 can activate the adenylyl cyclase/cAMP/CREB pathway in NSCLC [17], consistent with Bernhard et al.’s revelation that the ADRB2/CREB and NGF-BDNF/Trk pathways are promising targets for pancreatic cancer treatment [18]. Another research demonstrated the functional interactions between ADRB2 and VEGFR2 [19]. Supposedly, ADRB2 signaling plays a critical role in promoting cancer progression in multiple malignant cancers, including liver cancer [20], prostate cancer [21], and breast cancer [22]. In this study, we found that treatment with VEGFR2-TKIs resulted in upregulated ADRB2 expression in NSCLC cells. We used propranolol, a widely applicable cardiovascular drug that has been utilized to antagonize the β-adrenergic receptor for more than 50 years [23], to explore the antitumor property of combining it with VEGFR2-TKIs in NSCLC.

In this investigation, we targeted ADRB2 to enhance VEGFR2-TKI therapy in NSCLC. Our findings provided evidence for the combinatory use of VEGFR2-TKIs and propranolol to treat NSCLC in clinical practice.

## Results

### VEGFR2-TKI treatments resulted in upregulated ADRB2 expression in NSCLC

Anti-angiogenic therapy is vital in the treatment of NSCLC. Apatinib and anlotinib are oral anti-angiogenic drugs that target VEGFR2. However, anti-angiogenic therapy can cause adaptive resistance that leads to treatment failure; blocking the VEGFR signaling pathway could give rise to other angiogenic signaling pathway activities [24].

**Fig. 1:**
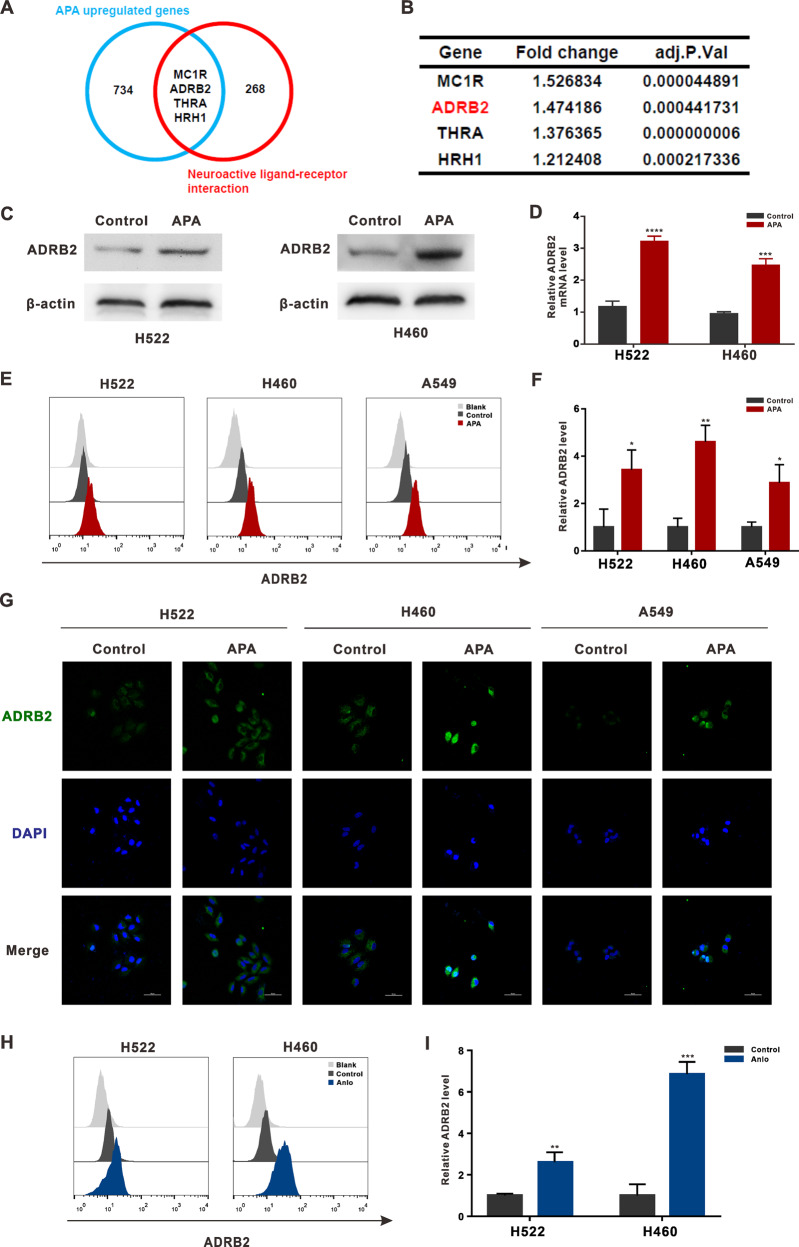
ADRB2 expression is upregulated by VEGFR2-TKIs treatment in NSCLC. **A** The overlapping analysis of apatinib upregulated genes and neuroactive ligand associated genes. **B** The results of the overlapped genes in RNA-seq data. **C** H522, H460, and A549 cells were treated with apatinib (20 μM) for 48 h. Western blotting was used to analyze the ADRB2 protein level. **D** RT-PCR was used to analyze the expression of ADRB2 mRNA. **E** The expression of ADRB2 was demonstrated by flow cytometry. **F** Statistical analysis of flow cytometry. **G** Representative images of immunofluorescent staining for ADRB2. **H** H522 and H460 cells were exposed to vehicle or anlotinib. The expression of ADRB2 was demonstrated by flow cytometry. **I** Statistical analysis of flow cytometry. (*P < 0.05, **P < 0.01, ***P < 0.001, ****P < 0.0001, compared with control).

To identify the VEGFR2-resistance mechanism, RNA-sequencing was used to examine differential gene expression. Data analyses revealed an increase in the expression of 734 genes after treatment with apatinib (Fig.1A and Table S1).

We previously demonstrated how adrenergic stress impacted the neovascularization of lung cancer [25]. Using that demonstration, we identified four intersection genes between upregulated genes and nerve-related receptors: MC1R, ADRB2, THRA, and HRH1 (Fig. 1A and B).

ADRB2 is a vital part of the sympathetic nervous system that supposedly plays a key role in tumor progression [26]. Given the suggestion that ADRB2 is associated with resistance to sorafenib in hepatoma carcinoma cells [20], we evaluated ADRB2 levels in the following research. RT-PCR revealed an increase in the ADRB2 gene expression in both H460 and A549 cells after treatment with apatinib (Fig. 1D), all consistent with Western Blot-determined protein levels (Fig. 1C). Flow cytometry confirmed the increased ADRB2 expression in the membrane post-treatment with apatinib (Fig. 1E). Immunofluorescence staining also showed significant cytoplasmic ADRB2 expression in apatinib-treated H460 and H522 cells compared to the control group (Fig. 1G).

To determine if the changes in ADRB2 expression were VEGFR-TKI-instigated, we also treated H522 and H460 cells with anlotinib, an inhibitor of VEGFR2. ADRB2 was also upregulated after treatment with anlotinib (Fig. 1F). Because ADRB2 expression and the therapeutic effect of apatinib correlated positively (Fig. S1), our data prove that treating NSCLC cells with VEGFR2-TKIs significantly increased ADRB2’s activity.

### ADRB2 activation reduced NSCLC cell sensitivity to VEGFR2-TKIs

**Fig. 2:**
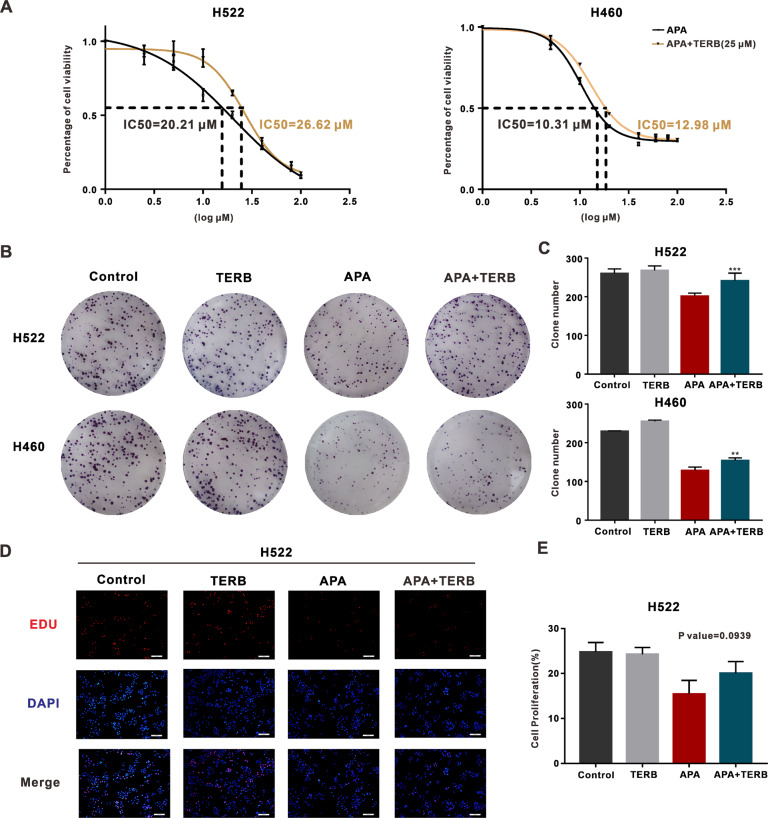
ADRB2 agonist terbutaline (TERB) promoted apatinib resistance in NSCLC. **A** H522 and H460 cells were exposed to vehicle or TERB (25 μM). IC50 of apatinib was measured with CCK8 assay. **B** Pictures of colony formation in H522 and H460 cells treated with vehicle, TERB, apatinib, or both modalities. **C** The clone numbers in four groups. **D** Representative images of EdU (EdU labeled with red, nuclei with blue). **E** The percentages of positive cells in four groups. (**P < 0.01, ***P < 0.001, NS not significant, compared with apatinib).

In our evaluation of whether the activation of ADRB2 signaling by terbutaline (TERB), an ADRB2 agonist, was involved in NSCLC resistance to apatinib therapy, terbutaline increased the IC50 of apatinib – 26.62 μM vs. 20.21 μM in H522 cells and 12.98 μM vs. 10.31 μM in H460 cells compared to the vehicle group (Fig. 2A). Terbutaline also increased the number and size of cell clone formations in the presence of apatinib (Fig. 2B).

EdU (5-ethynyl-2′-deoxyuridine), a thymine nucleoside analog that can replace thymine (T) in the replication of DNA during cell proliferation, can help in the rapid detection of DNA replication and cell proliferation [27]. Combining Apatinib and propranolol displayed higher EdU-positive cell levels than treatment with apatinib alone (Fig. 2D, E). These results suggest that (TERB) decreased the efficacy of apatinib during cell proliferation in NSCLC cell lines.

### Combining VEGFR2-TKIs with propranolol synergistically decreased cell proliferation in NSCLC

To further probe the role of induced ADRB2 activity in NSCLC cell line resistance to apatinib, we inhibited ADRB2 signaling activity with an ADRB2 antagonist, ICI118551. The result was the fall in apatinib’s IC50 from 35.14 to 12.42 μM in H522 cells and from 13.58 to 3.36 μM in H460 cells (Fig. S2).

**Fig. 3:**
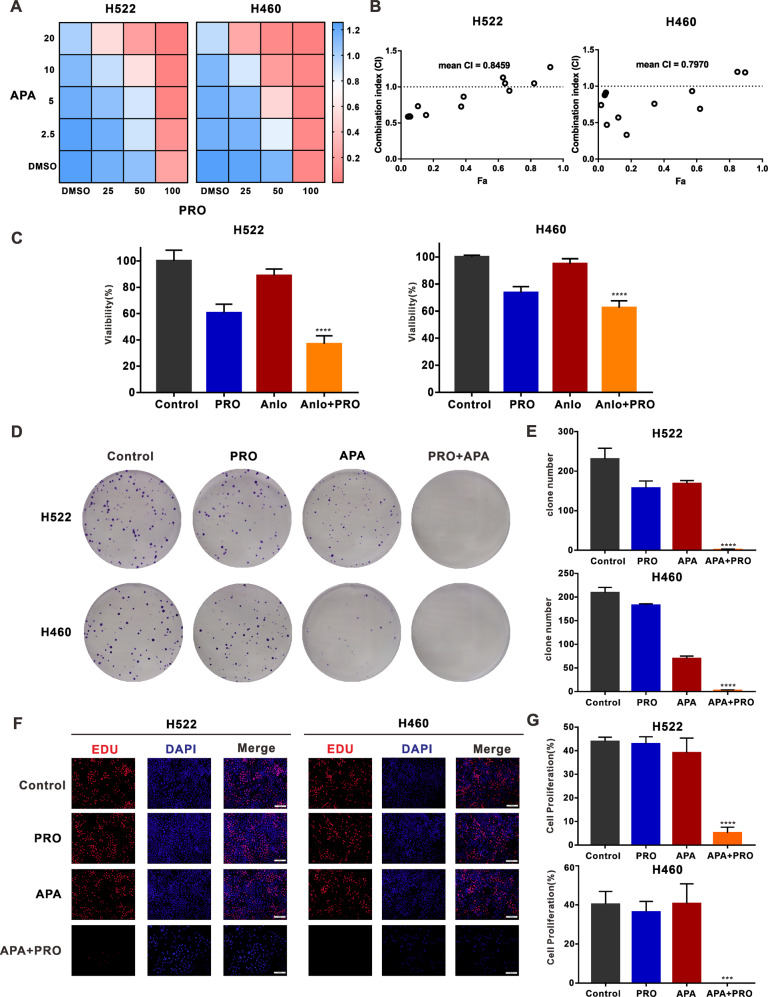
ADRB2 antagonist propranolol combined with apatinib inhibits cell proliferation. **A** H522 and H460 cells were exposed to various concentrations of apatinib and propranolol for 48 h, and CCK8 assay was used to detect the cell viability. **B** Combination index (CI) was determined by CompuSyn software. **C** the CCK-8 assay detected the viability of H522 and H460 cells after anlotinib and propranolol treatment. **D** Representative images of H522 and H460 clones treated with DMSO, propranolol, apatinib, or both modalities. **E** The clone numbers in four groups. **F** Representative images of EDU (Red, EdU marked proliferative cells, blue, Hoechst 33342 marked nuclei). **G** The percentages of positive cells in four groups. (***P < 0.001, ****P < 0.0001, compared with apatinib).

Propranolol, an antagonist of ADRB2, was used to assess apatinib’s efficacy. The CCK-8 assay performed to detect the activity of NSCLC cells treated with different concentrations of apatinib and propranolol (Fig. 3A and Fig. S3) revealed that combining the two substances was most effective in H522 and H460 cell lines (Fig. 3A). A plot of the CI values of the combination versus cellular fraction affected (Fa) showed a strong synergy between apatinib and propranolol (Fig. 3B).

Because of its wide range of clinical applications, we used propranolol further in the follow-up experiments. The CCK-8 assay confirmed the synergy between anlotinib and propranolol as a combinatory treatment (Fig. 3C). Combined treatment with apatinib and propranolol decreased the number and size of cell clones compared to either drug alone (Fig. 3D). This treatment approach also notably reduced EdU-positive cells compared to apatinib treatment alone (Fig. 3F). These data demonstrate that the ADRB2 antagonist helped determine the efficacy of VEGFR2-TKIs in NSCLC cell lines.

### Combining VEGFR2-TKIs with propranolol **promoted** apoptosis and increased ROS levels

**Fig. 4:**
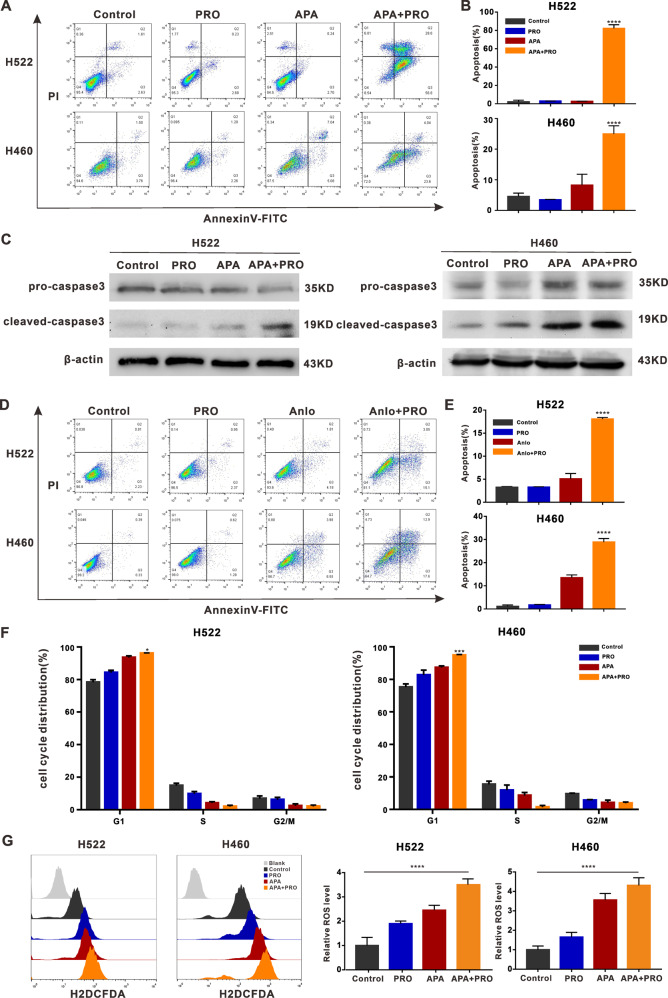
ADRB2 antagonist propranolol combined with apatinib can promote apoptosis in NSCLC. **A** H522 and H460 cells were treated with/without apatinib (20 μM) and propranolol (50 μM) for 48 h. The apoptosis index was detected by flow cytometry. **B** The percentage of apoptotic cells in H522 and H460 cells. **C** Western blot revealed expression level of caspase3 and cleaved-caspase3 protein. **D** H522 and H460 cells were treated with/without anlotinib (2.5 μM) and propranolol (50 μM) for 48 h. The apoptosis index was detected by flow cytometry. **E** The percentage of apoptotic cells in H522 and H460 cells. **F** Flow cytometry showed the proportion of cells at different stages of cell cycle. The percentages of cell cycle phases in four groups. **G** Flow cytometry was used to analyze ROS levels in H522 and H460 cells. (*P < 0.05; **P < 0.01; **P < 0.001; ****P < 0.0001, compared with apatinib).

To explore the main mechanism for inhibiting tumor cell proliferation in the VEGFR2-TKIs and propranolol combination treatment approach, we used Annexin-V-FITC and propidium iodide to detect cell apoptosis. As shown in Fig. 4A, the treatment approach significantly induced H460 and H522 cell apoptosis.

Caspase3 is a key enzyme in the execution of apoptosis, and the detection of cleaved-caspase3 is considered a reliable marker for apoptotic cells [28]. Western blotting analysis revealed that cells treated with combined apatinib and propranolol exhibited higher cleaved-caspase3 expression than those treated with apatinib alone (Fig. 4C). Similarly, flow cytometry analyses showed that apoptotic cell percentage markedly increased after treatment with combined anlotinib and propranolol compared to treatment with anlotinib alone (Fig. 4D).

To assess the impact of combing VEGFR2-TKIs and propranolol on cell cycle arrest, we treated cells with combined apatinib and propranolol for 48 h, which increased the proportion of cells in the G1 phase (Fig. 4F).

Because toxic levels of ROS reportedly induce cell cycle arrests and apoptosis [29], we measured ROS levels with flow cytometry using the fluorescent probe H_2_DCFH-DA. Apoptosis and ROS significantly increased in NSCLC cells treated with combined apatinib and propranolol compared to treatment with apatinib alone (Fig. 4G).

### Combining VEGFR-TKIs with propranolol inhibited the ADRB2/CREB signaling pathway and reduced PSAT1 expression

**Fig. 5:**
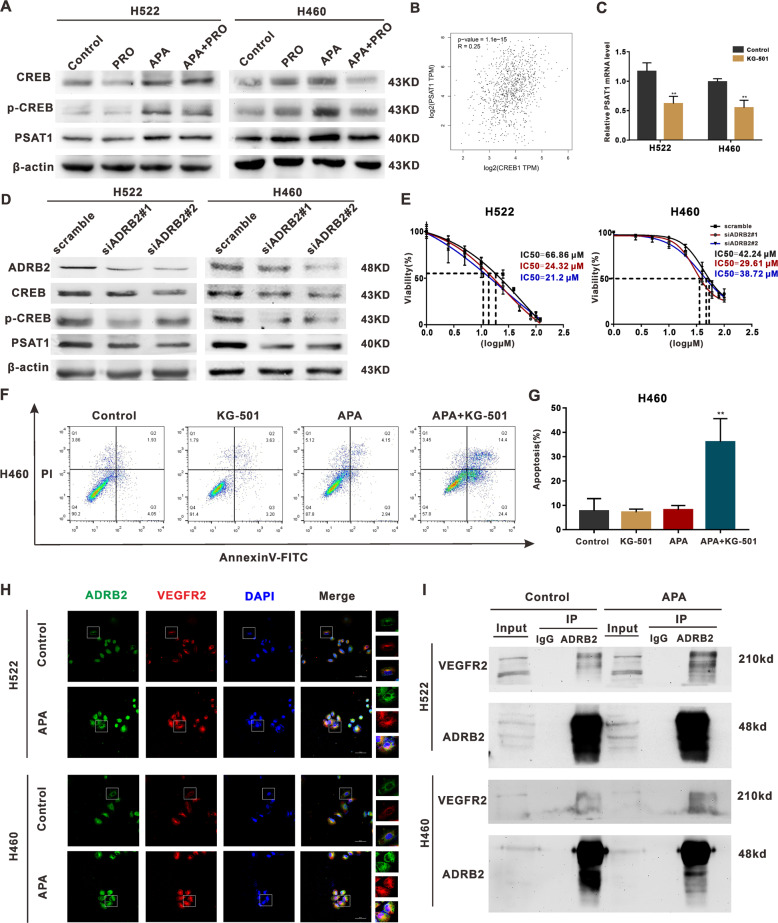
The mechanism of apatinib resistance in NSCLC cells. **A** H522 and H460 cells were treated with or without apatinib (20 μM) and propranolol (50 μM) for 48 h. The relative protein level of CREB, p-CREB, PSAT1 was detected through Western blot. **B** A positive correlation between CREB and PSAT1 expression in lung cancer according to TCGA database. **C** qRT-PCR analysis of PSAT1 mRNA levels in H522 and H460 cells after KG-501 treatment. **D** Western blot analysis of CREB, p-CREB, PSAT1 expression in H522 and H460 cells after ADRB2 knockdown. **E** H522 and H460 cells were transfected with indicated siRNAs for 48 h, and the samples were exposed to different concentrations of apatinib. IC50 of apatinib was measured with CCK8 assay. **F** H460 cells were treated with/without apatinib (20 μM) and KG-501 (25 μM) for 48 h. The apoptosis index was detected by flow cytometry. **G** The percentage of apoptotic cells in H522 and H460 cells. **H** Representative co-localization images stained with ADRB2 (green) and VEGFR2 (red) in H522 and H460 cells. **I** Anti-ADRB2 antibodies immunoprecipitated endogenous ADRB2 and Co-IP VEGFR2 by Western blot analysis. (**P < 0.01, compared with vehicle).

Supposedly, ADRB2 upregulates CREB expression [17]. In line with previous studies, antagonizing ADRB2 and knocking down ADRB2 lessened the induction of phosphorylated CREB (Fig. 5A and D). Per Wei et al. [30], PSAT1 activation is crucial to the resistance to sorafenib in HCC. Consistent with that claim, we found that combining apatinib with propranolol reversed the apatinib-induced increase in PSAT1 (Fig. 5A). ADRB2 knockdown also saw PSAT1 expression decrease (Fig. 5D). with these findings and because the analysis of the TCGA database yielded a positive correlation between PSAT1 and CREB expressions (Fig. 5B), we inferred that the CREB/PSAT1 signaling pathway plays a key role in the development of resistance to VEGFR2-TKIs.

To determine ADRB2/CREB signaling’s ability to regulate PAST1 expression, we inhibited CREB with KG-501 and found that KG-501 significantly suppressed PSAT1 mRNA levels (Fig. 5C). As expected, knocking down ADRB2 also resulted in reduced IC50s for apatinib (Fig. 5E). The apoptosis of H460 cells treated with a combination of apatinib and KG-501 increased considerably (Fig. 5F). These results demonstrate that activating the ADRB2/CREB/PSAT1 pathway suppressed VEGFR2-TKIs’ efficacy.

### VEGFR2-TKI treatment enhances VEGFR2 and ADRB2 interaction

Previous studies have suggested that the oligomeric complexes between VEGFR2 and ADRB2 can be produced in the cell membrane and intracellular endosomes through bioluminescence resonance energy transfer and NanoLuc luciferase-tagged VEGFR2 [19] and that treatment with ICI 118,551 could inhibit isoprenaline-induced VEGFR2 upregulation [31]. Therefore, we speculated that treatment with apatinib could enhance the interaction between ADRB2 and endogenous VEGFR2. To test this hypothesis, we first co-stained NSCLC cells with anti-VEGFR2 and anti-ADRB2 antibodies via immunofluorescence and observed the colocalization of VEGFR2 and ADRB2 proteins through confocal microscopy (Fig. 5H). Coimmunoprecipitation (Co-IP) assays showed the existence of an interaction between ADRB2 and VEGFR2. This interaction in apatinib-treated cells was further enhanced and observed (Fig. 5H and I).

### Combining VEGFR2-TKI with propranolol slowed down tumor growth

**Fig. 6:**
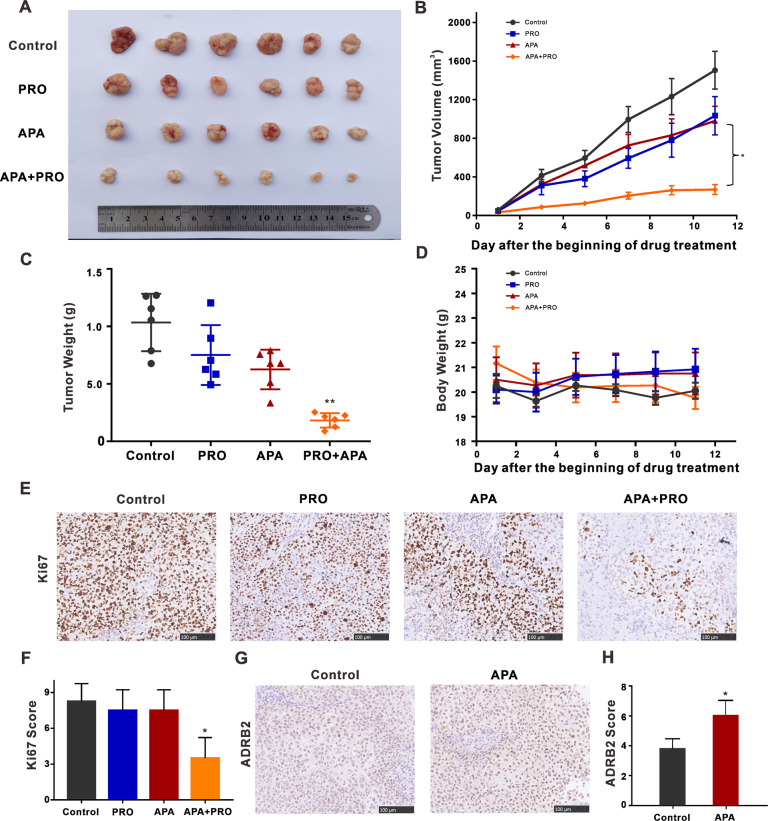
Combined apatinib and propranolol treatment significantly decreases H460-xenograft growth in vivo. **A** Mice were grouped for treatment with 0.9% normal saline group, apatinib (50 mg/kg/day), propranolol (40 mg/kg/day), or both modalities by intragastric injection during the study. Tumors were dissected from nude mice. The inset picture shows the tumor in the respective group. **B** The growth curve of tumor volume was shown. The data represent mean tumor volumes ± SEMs. **C** Tumor weight of four groups was compared on the last day. The tumor volumes and weights were presented as means ± SDs (*n* = 6 for each group). **D** Body weight changes of mice during drug administration. The data were presented as the mean of tumor volumes ± SEMs. **E** Immunohistochemistry staining of Ki67 of tumor sections in each group. **F** IHC score of Ki67 staining. Pictures were captured at a light microscope with ×20 magnifications. (compared with apatinib) **G** ADRB2 immunohistochemical staining of lung tumors in each group. **H** IHC score of ADRB2 staining. Pictures were captured at a light microscope with ×20 magnifications. (*P < 0.05, **P < 0.01, compared with control).

To evaluate the anti-tumor capabilities of combined VEGFR2-TKI and propranolol in vivo, we established a subcutaneous transplantation tumor model using BALB/c nude mice. Mice with palpable tumors were divided into four groups randomly: control, apatinib, propranolol, and apatinib and propranolol combination (*n* = 6). For mice receiving apatinib, each was given 50 mg/kg of the drug by gavage daily throughout the test, and for those given propranolol, each received 40 mg/kg by gavage daily for the entirety of the experiment too. The tumor volume of mice in the combined substance treatment group was significantly smaller than that for the single substance treatment groups (Fig. 6A and C). Combined apatinib and propranolol treatment resulted in a more efficient tumor growth delay than the use of each drug alone. Bodyweight or signs of toxicity in treatment groups were not drastically different (Fig. 6D). Ki67 is a cellular marker of proliferation whose expression was decreased by the combination treatment after IHC staining, indicating that the combinatory therapy suppressed NSCLC cell proliferation (Fig. 6E). Per analyses of tumor tissues by immunohistochemistry (IHC), apatinib treatment enhanced ADRB2 expression (Fig. 6F). These data proved further that treatment with VEGFR2-TKIs increased ADRB2 expression in NSCLC.

## Discussion

In recent years, anti-angiogenesis agents, including apatinib and anlotinib, targeting the VEGFR2 pathway have shown significant efficacy in a variety of cancers. Apatinib is one of the most promising antiangiogenic target drugs; in registered phase I and II clinical trials [32], it has shown favorable therapeutic features and tolerable toxicity. Anlotinib is another VEGFR2 TKI used as third-line therapy for advanced NSCLC [33]. However, short-lived therapeutic response and drug resistance often lead to treatment failure. Finding an effective combination therapy to enhance VEGFR2-TKI treatments is a reasonable strategy that could solve these issues. With our findings in this study, we concluded that VEGFR2-TKI treatments alone failed to achieve the expected outcome because of the increased activated β_2_-adrenergic receptor (ADRB2) signaling and the treatment-induced upregulation of VEGFR2 expression. While these results shed a light on the possible important role of adrenergic receptors in antitumor therapeutic resistance, they also show that the impact of VEGFR2-TKIs could be enhanced through targeting ADRB2 signaling.

β-adrenergic signaling regulates tumor development by controlling various downstream pathways [34], and it can cause stress response through the sympathetic nervous system [35]. According to epidemiologic studies, stress-related factors have an adverse effect on tumor outcomes [36]. All three β-adrenergic receptors are expressed in lung cancer-derived cells, but ADRB2 is expressed at a higher level than the others. Compared with the adjacent normal airway or alveolar epithelia, ADRB2 enhances the immunoreactivity of tumor cells [37]. The immunoreactivity of β_1_-adrenergic receptors (ADRB1) has been detected but not overexpressed in tumor cells, suggesting that ADRB2 plays a leading role in lung cancer compared with other beta-adrenergic receptors. In this study, we showed a positive relationship between the IC50 value of apatinib and ADRB2 expression in the four different NSCLC cell lines tested, which possibly confirms the association between sensitivity to apatinib and ADRB2 expression in NSCLC; however, the difference was not statistically significant (*P* = 0.0801), almost certainly due to the small sample size.

In literature, ADRB2 activation is linked to increased lung cancer risk and may be involved in lung cancer development [38, 39]. Consistent with that observation, we established here that the ADRB2 agonist, TERB, stimulated NSCLC cell proliferation. Moreover, the combined treatment reduced the effectiveness of apatinib on tumor cells. Because ADRB2 knockdown significantly increased the IC50 values of apatinib in H522 and H460 cells, we combined the ADRB2 antagonists, ICI118551, and propranolol, with apatinib, separately. Both combinations enhanced the antitumor action of apatinib, even if propranolol is preferred due to extensive clinical application and palpable effects. Because the combination therapy of VEGFR2-TKIs and propranolol-induced apoptosis significantly increased with the level of ROS, we assumed that the overproduction of ROS triggered cell apoptosis. Evaluating the impact of VEGFR2-TKIs and propranolol combination therapy in vitro and in vivo reminded us that the stress levels of patients could be associated with sensitivity to VEGFR2-TKIs, and psychological rehabilitation intervention improves the clinical efficacy of anti-VEGFR2 therapy in NSCLC patients. Banerjee et al. revealed that stress reduction significantly reduces the growth of NSCLC xenografts in mice compared with mice maintained under controlled conditions [40], which is similar to the finding that enhanced VEGFR2 expression significantly increased tumors in the mouse model of repeated social defeat stress (RSDS) [41], suggesting that chronic stress contributes to tumor progression, invasion, and metastasis and might be associated with VEGF2 secretion.

Existing studies have found that a dimer complex can be formed between ADRB2 and VEGFR2 [19]. We showed that the VEGFR2 and ADRB2 interaction regulated downstream signaling in NSCLC cells and apatinib treatment significantly enhanced the complex formations between ADRB2 and VEGFR2. In addition, NSCLC cells treated with combined apatinib and propranolol had substantially decreased vasculogenic mimicry formation and number of migrating cells compared to the single-drug groups (Figs. S4 and S5). These experiments indicate that blocking the activity of ADRB2 could reduce tumor angiogenesis and migration.

We have also demonstrated that ADRB2 signaling regulates the activity of PSAT1 through CREB phosphorylation. PSAT1, an enzyme that catalyzes serine biosynthesis, is highly upregulated in NSCLC tissues [42]. Previous studies have revealed that PSAT1 promotes cell cycle progression, cell proliferation, and tumorigenesis [43]. Supposedly, targeting key enzymes in the serine synthesis pathway (PHGDH, PSAT1, and PSPH) is an effective approach to overcoming resistance to sorafenib in HCC [30]. Here, we showed that ADRB2 regulated PSAT1 expression but ADRB2 treatment with propranolol combined with apatinib elevated ROS levels and induced NSCLC cell apoptosis. We reasonably speculate that resistance to VEGFR2-TKIs is associated with serine metabolism; this, we expect, should be a new research direction in NSCLC.

In summary, treatment with anti-VEGFR2 inhibitors induced ADRB2 upregulation, with the feedback-activated ADRB2 signaling causing therapeutic resistance to VEGFR2-TKIs. However, blocking the ADRB2/CREB/PSAT1 axis enhanced the sensitivity of NSCLC to VEGFR2-TKIs and provided a novel promising combinatory approach to enhance the antitumor effect of VEGFR2-TKIs in NSCLC.

## Materials and methods

### Cell lines and reagents

Human NSCLC cell lines H522, H460, A549, and H292 were purchased from the American Type Culture Collection (ATCC). All cell lines were cultured in RPMI 1640 medium (Gibco) containing 10% fetal bovine serum (Gibco) at 37 °C in a 5% CO_2_ incubator. Apatinib (Selleckchem) and anlotinib (Selleckchem) were dissolved in dimethylsulfoxide (DMSO) and stored at −80 °C (20 mM, stock solution). Propranolol (Selleckchem) was dissolved in DMSO and stored at −80 °C (50 mM, stock solution). KG501 (Selleckchem) was dissolved in DMSO and stored at −80 °C (25 mM, stock solution).

### Quantitative RT-PCR

Total RNA was extracted using the Omega Total RNA Kit (R6834, OMEGA). HiScript III RT SuperMix (+gDNA wiper) (r323-01, Vazyme) reagents were used to synthesize cDNA. Quantitative reverse transcription-PCR (qRT-PCR) using the AceQ Universal SYBR qPCR Master Mix (a111-02, Vazyme) was performed by a StepOne Plus Real-Time PCR System (Applied Biosystems). PCR conditions were as follows: 95 °C for 30 s, then 40 cycles of 95 °C for 15 s, and a melting curve analysis at 95 °C for 15 s, 60 °C for 30 s, and 95 °C for 15 s. The following sequences were shown as followed: ADRB2 forward primer 5′-GCCTGTGCTGATCTGGTCAT- 3′; ADRB2 reverse primer 5′-AATGGAAGTCCAAAACTCGCA-3′; PSAT1 forward primer 5′-GGGAATTGCTAGCTGTTCCAG-3′; PSAT1 reverse primer 5′-TCAGCACACCTTCCTGCTTT-3′; β-actin forward primer 5′-CCTGGCACCCAGCACAAT-3′; β-actin reverse primer 5′-GGGCCGGACTCGTCATAC-3′. The experiments were repeated three times.

### Western blot analysis

Cells were collected and washed twice in PBS and lysed in NETN buffer (100 mM NaCl, 0.5 mM EDTA, 20 mM Tris-HCl [pH 8.0] and 0.5% Nonidet P-40). Cell lysate was mixed with SDS sample buffer and denatured at 100 °C for 8 min. Samples were electrophoretically separated on 10–12% SDS polyacrylamide gel. After electrophoresis, the protein was transferred to polyvinylidene difluoride (PVDF) membranes, then closed with 5% skim milk for 1 h. Following incubation overnight at 4 °C with primary antibodies: anti-ADRB2 (ab61778, Abcam), anti-CREB (#9197, Cell Signaling Technology) and anti-phospho-CREB (#9198, Cell Signaling Technology), anti-PSAT1 (A6707, ABclonal), anti-caspase3 (AC030, Beyotime Biotechnology), anti-cleaved caspase-3 (#9661, Cell Signaling Technology) and anti-β-actin (AC00, ABclonal). The membranes were washed three times with TBST and incubated with HRP secondary antibodies for 1 h at room temperature. Finally, the PVDF membranes were detected by enhanced chemiluminescence.

### Immunoprecipitation

Cells were collected and washed twice in PBS and lysed in NETN buffer. After centrifuge for 25 min, the supernatant was obtained and mixed with anti-ADRB2 antibody (sc-81557, Santa Cruz Biotechnology) and Protein A/G (Santa Cruz Biotechnology) overnight. The immune complexes were collected by centrifugation and washed five times with NETN buffer. The supernatant was removed after the last wash. The immune complexes were added 1× SDS loading buffer and boiled for 10 min at 100 °C. The samples were detected by Wb. The primary antibody was as follows: anti-VEGFR2 (#2479, Cell Signaling Technology) and anti-ADRB2 (ab61778, Abcam).

### CCK8 assay

Tumor cells were cultured at a density of 5 × 10^3^ cells/well in 96-well plates. After adhering to the wall, cells were treated with different concentrations of apatinib (0, 2.5, 5, 10, 20 μM) and propranolol (0, 25, 50, 100 μM) for 48 h. Cell viability was detected by the CCK-8 kit (biosharp) according to the instructions. The experiments were repeated three times.

### Colony-formation assay

H522 and H460 cells were planted at a density of 400 cells per well in six-well plates. After attachment, the cells were treated with apatinib (20 μM) and propranolol (50 μM) for 48 h. The medium was replaced by drug-free medium to end drug exposure and cells were cultured for two weeks. The culture was terminated when a single clone contained more than 50 cells. Cell clones were fixed and stained with crystal violet.

### RNA interference

Transfection of siRNA was performed using Lipofectamine RNAiMAX reagent (Invitrogen) for 48 h following the manufacturer’s instructions. The siRNA sequences were shown as followed:

ADRB2 siRNA#1, 5-GTACTGTGCCTAGCGATAA-3′

ADRB2 siRNA#2, 5′-CATCCTCCTAAATTGGATA-3′.

### Immunofluorescence

H460 and H522 cells were planted on the cell slides in the 24-well plates at a density of 5 × 10^3^ and treated with apatinib (20 µM) or vehicle for 48 h. The cells were rinsed twice with PBS and fixed with 4% paraformaldehyde for 15 min. The cells were blocked with 5% BSA for 1 h at room temperature. The cells were stained with primary antibodies and appropriate fluorescent secondary antibodies. The samples were observed by the fluorescence microscope.

### EdU assay

H522 and H460 cells (8 × 10^3^) were plated in 96-well plates. After attaching overnight, cells were treated with apatinib (20 μM) and propranolol (50 μM) for 48 h. Subsequently, the cells were incubated with EdU (1:1000) for 2 h. The samples were washed with PBS and then fixed with 4% paraformaldehyde for 30 min. The cells were stained using the EdU assay. Nuclear was stained with Hoechst 33342. Images were acquired from the fluorescence microscope.

### Cell cycle analysis

Cells were cultured in the six-well plate. Allowed to attach overnight and treated with apatinib (20 μM), propranolol (50 μM), or the combination for 48 h. Cells were then harvested and fixed in 70% ethanol at 4 °C overnight. After cells were washed in PBS three times, Propidium iodide (PI) and RNase were added to the cells in the dark for 1 h at 4 °C. Cell cycle distribution was detected by flow cytometry. The ModFit software was used for data analysis.

### Flow cytometry

Cells were treated with apatinib (20 μM) for 48 h. Cells were then harvested and washed twice with PBS. Cells were stained with ADRB2 antibody (13096-1-AP, Proteinech) and then incubated at room temperature in the dark for 1 h. ADRB2 mean fluorescence intensity was detected by flow cytometry.

### Apoptosis analysis

Cells were cultured in six-well plate. Allowed to attach overnight and treated with apatinib (20 μM), propranolol (50 μM) or the combination for 48 h. Cells were then harvested and washed twice with PBS. Then, 5 μL Annexin V-FITC and 10 μL PI were added and cells were incubated at room temperature in the dark. Cell apoptosis was detected by flow cytometry.

### RNA sequencing

Total RNA was collected from A549 cells which were treated with or without apatinib (20 μM) by Total RNA Kit I reagent (OMEGA, R6834). RNA sequencing was performed by Shanghai Majorbio Bio-Pharm Technology Co., Ltd. The differential gene expression analysis and biological pathway analysis were conducted.

### Tube formation

The Matrigel (50 μM per well, BD Biosciences) was added to the well in a 96-well plate with a precooled pipette. The plate was then put in the incubator a 37 °C for 30 min. Cells were resuspended in the medium containing vehicle, apatinib, propranolol, or the combination of apatinib and propranolol. Subsequently, the cell suspension (8 × 10^3^) was seeded onto the Matrigel-coated wells. After 4 h, tubular structures were observed and pictured under the light microscope.

### Transwell migration assay

H460 cells were pretreated with vehicle, propranolol, apatinib, and the combination for 48 h and then added to the upper chamber in 250 μL serum-free medium. Five hundred microliters of medium containing 10% fetal bovine serum were added to the lower chamber. The chambers were incubated for 48 h at 37 °C. Subsequently, the cells were fixed and stained with crystal violet.

### Animal experiments

Female BALB/c nude mice of 4–6 weeks were purchased from Changzhou Cavens Laboratory Animal Co., Ltd and were bred in Tongji Medical College, Huazhong University of Science and Technology (HUST). The animal experiments were reviewed and approved by the Medical Ethical Committee of HUST. Mice anaesthetized with nembutal were inoculated with the cell suspension of H460 cells (5 × 10^7^ cells/mL) in 100 μL of serum-free media into the right fore limbs. Mice with palpable tumors were randomly divided into four groups (6 mice/group), the normal saline group, the apatinib group (50 mg/kg/day), the propranolol group (40 mg/kg/day), and the combination of apatinib and propranolol group. Mice were treated by gavage every day. Tumor growth and body weight were measured every other day, and the tumor volume did not exceed 2000 mm^3^. Mice were euthanised by cervical dislocation. Tumor volume was calculated using the formula: length × width^2^ × 0.5.

### Statistics

Samples or animals were assigned to treatment conditions randomly and data analysis was conducted in a blinded fashion. Each experiment was repeated at least in triplicate and results were presented as mean ± SD unless otherwise described. Student’s *T*-test (unpaired, two-tailed) and analysis of variance (ANOVA) were used to test for statistical significance by GraphPad 7.0 Prism software. *P* < 0.05 was considered statistically significant.

## Supplementary information

Author contribution statement

Supplementary Table

Supplementary information

## Data Availability

For additional details, see the Supplementary material
