# Supplementary material for: Targeting ADRB2 enhances sensitivity of non-small cell lung cancer to VEGFR2 tyrosine kinase inhibitors
Source: Cell Death Discov. 2022 Jan 24;8:36. doi: 10.1038/s41420-022-00818-8 (PMC8786837; doi:10.1038/s41420-022-00818-8)
Supplement: Supplementary file 1 — Author contribution statement [file 41420_2022_818_MOESM1_ESM.docx]

**Author contribution statement**

G.W, Z.L, J.W, and Y.X conceived and designed the study.

Y.X, J.W, J.T, X.J, J.Y, R.R, H.X, B.X and X.W conducted experiments and analyzed the data.

J.W, X.Z and G.W provided advice and technical assistance.

Y.X, J.W and G.W wrote the manuscript.

G.W supervised the study.

All authors reviewed and approved the final manuscript.
