# Supplementary material for: Targeting ADRB2 enhances sensitivity of non-small cell lung cancer to VEGFR2 tyrosine kinase inhibitors
Source: Cell Death Discov. 2022 Jan 24;8:36. doi: 10.1038/s41420-022-00818-8 (PMC8786837; doi:10.1038/s41420-022-00818-8)
Supplement: Supplementary file 3 — Supplementary information [file 41420_2022_818_MOESM3_ESM.docx]

**Supplementary information**

**Table S1.** Gene lists for Figure 1a


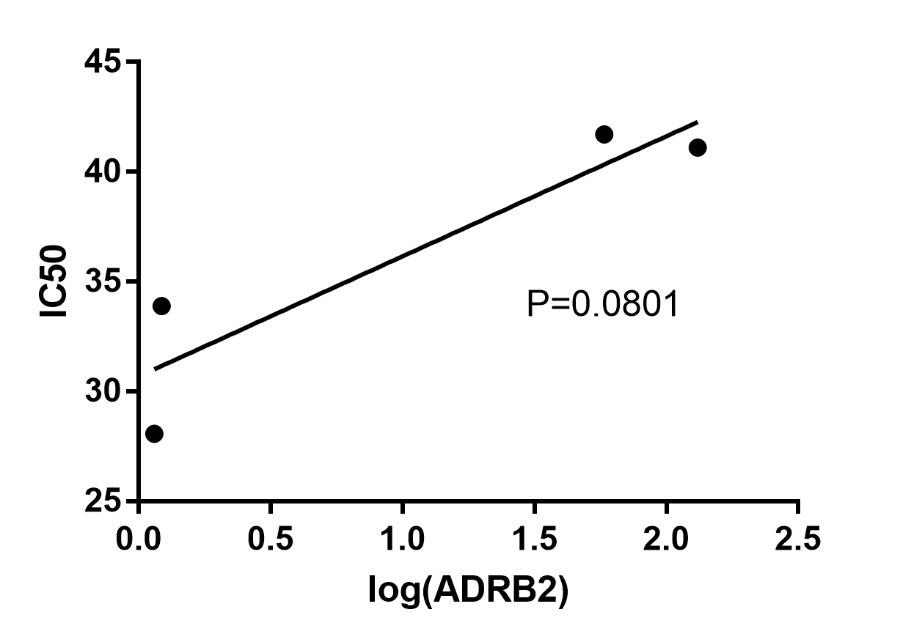


**Figure S1.** Correlation between basic expression of ADRB2 and IC50s of apatinib


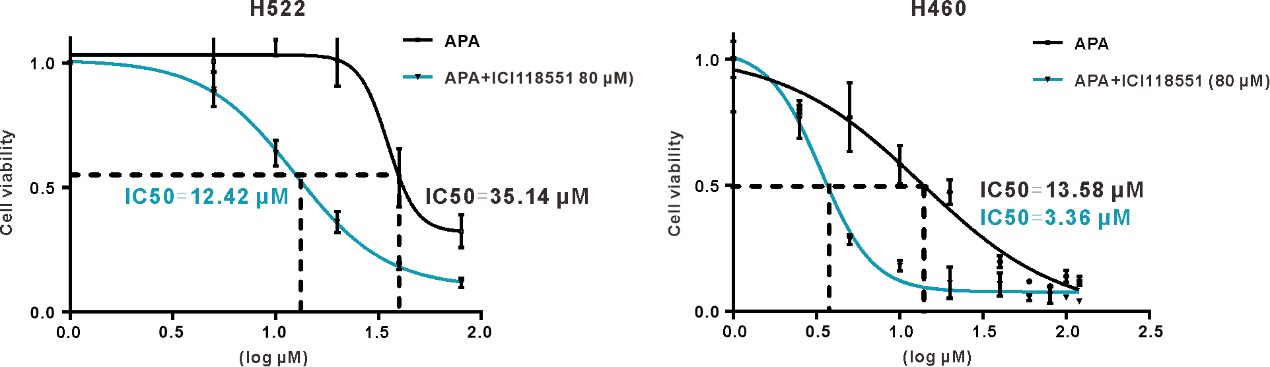


**Figure S2.** H522 and H460 cells were exposed to vehicle or ICI118551 (25 μM). IC50 of apatinib was measured with CCK8 assay.


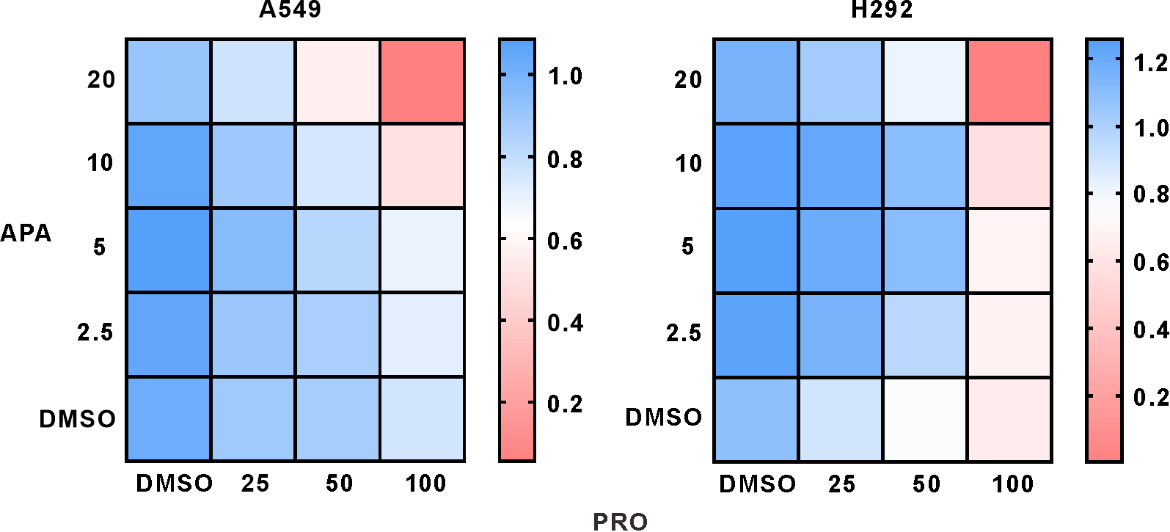


**Figure S3.** A549 and H292 cells were exposed to various concentrations of apatinib and propranolol for 48 h, and CCK8 assay was used to detect the cell viability.

**
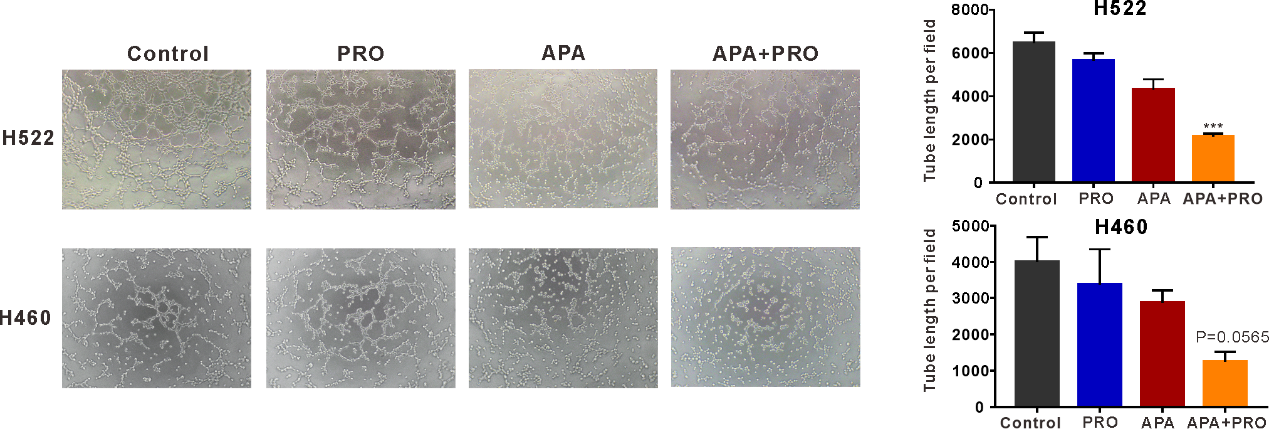
**

**Figure S4.** Pictures of tube formation ability in H522 and H460 cells treated with vehicle, propranolol, apatinib, or both modalities.


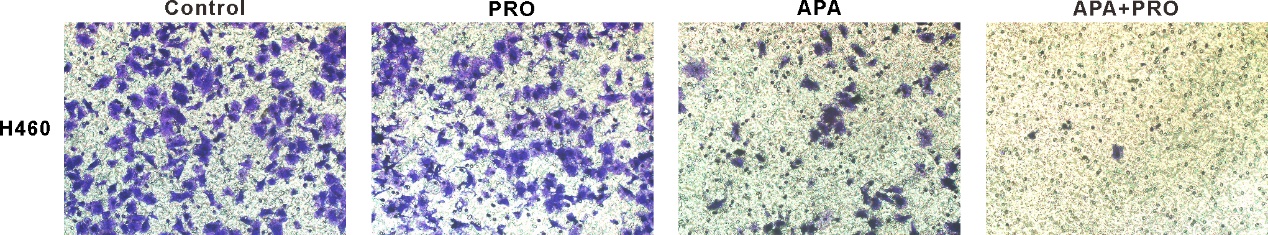


**Figure S5.** Pictures of transwell migration assay in H460 cells treated with vehicle, propranolol, apatinib, or both modalities.
